# 4,4′-Bis[2-(3,4-dibutyl-2-thienylethyn­yl)]biphen­yl

**DOI:** 10.1107/S1600536808036143

**Published:** 2008-11-13

**Authors:** Lei Liu, Na Liu, Wei Xu, Dao-Ben Zhu

**Affiliations:** a College of Chemical and Pharmaceutical Engineering, Hebei University of Science and Technology, Shijiazhuang, Hebei 050018, People’s Republic of China; b College of Bioscience and Biotechnology, Hunan Agricultural University, Hanan 410128, People’s Republic of China; c 106 Group, Institute of Chemistry, Chinese Academy of Sciences, Beijing 100080, People’s Republic of China

## Abstract

The mol­ecule of the title compound, C_40_H_46_S_2_, reveals *C*_*i*_ symmetry. An inversion centre is located at the mid-point of the C—C bond of the biphenyl unit; the asymmetric unit comprises one-half of the mol­ecule. The conjugated backbone is nearly planar, with a mean deviation of 0.041 Å.

## Related literature

For general background, see: Brad Wan *et al.* (2000 ▶); Cornil *et al.* (2001 ▶); Grosshenny *et al.* (1997 ▶); Huang & Tour (1998 ▶); Tour (1996 ▶). For related structures, see: Baudour (1972 ▶);  Charbonneau & Delugeard (1977 ▶); Domenicano *et al.* (1975 ▶); Robertson (1961 ▶). For the synthesis, see: Liu *et al.* (2005 ▶).
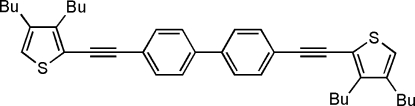


## Experimental

### 

#### Crystal data

C_40_H_46_S_2_
*M* *_r_* = 590.89
Triclinic, 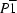

*a* = 9.2040 (18) Å
*b* = 9.3640 (19) Å
*c* = 10.582 (2) Å
α = 85.69 (3)°
β = 85.18 (3)°
γ = 69.41 (3)°
*V* = 849.7 (3) Å^3^
*Z* = 1
Mo *K*α radiation
μ = 0.18 mm^−1^
*T* = 293 (2) K
0.62 × 0.40 × 0.07 mm

#### Data collection

Rigaku R-AXIS RAPID IP diffractometer
Absorption correction: empirical (using intensity measurements) (*ABSCOR*; Higashi, 1995 ▶) *T*_min_ = 0.805, *T*_max_ = 0.992
3595 measured reflections
3595 independent reflections
2287 reflections with *I* > 2σ(*I*)

#### Refinement

*R*[*F* ^2^ > 2σ(*F* ^2^)] = 0.067
*wR*(*F* ^2^) = 0.241
*S* = 1.07
3595 reflections
190 parameters
H-atom parameters constrained
Δρ_max_ = 0.44 e Å^−3^
Δρ_min_ = −0.42 e Å^−3^

### 

Data collection: *RAPID-AUTO* (Rigaku, 2001 ▶); cell refinement: *RAPID-AUTO*; data reduction: *RAPID-AUTO*; program(s) used to solve structure: *SHELXS97* (Sheldrick, 2008 ▶); program(s) used to refine structure: *SHELXL97* (Sheldrick, 2008 ▶); molecular graphics: *SHELXTL* (Sheldrick, 2008 ▶); software used to prepare material for publication: *SHELXTL*.

## Supplementary Material

Crystal structure: contains datablocks 070712a, I. DOI: 10.1107/S1600536808036143/kp2189sup1.cif

Structure factors: contains datablocks I. DOI: 10.1107/S1600536808036143/kp2189Isup2.hkl

Additional supplementary materials:  crystallographic information; 3D view; checkCIF report

## Figures and Tables

**Fig. 1.**
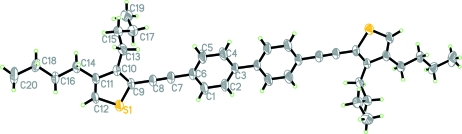
View of the title compound, showing the atom-labelling scheme and displacement ellipsoids at the 50% probability. To generate the molecule symmetry code -x, -y, -z + 2 is applied.

**Fig. 2.**
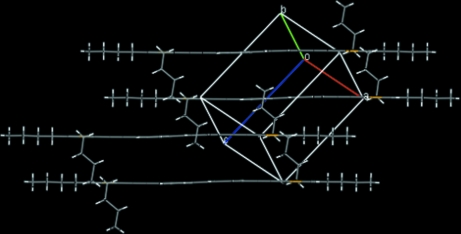
View of the packing mode along a axis of (I).

## supplementary crystallographic information

### Comment

The synthesis and characterization of nanometer-sized conjugated molecules of precise length and constitution are of widespread interest, which is due to their electroconductive, magnetic, and optical properties (Tour, 1996; Huang & Tour, 1998; Grosshenny *et al.*, 1997; Brad Wan *et al.*, 2000). Generally, crystal structure of a molecule is important for better understanding of its properties. Therefore, structures of oligothiophene single crystals have been reported. The field of molecular organic semiconductors is being revolutionized by the availability of ultrahigh purity single crystals that have allowed the demonstration of phenomena long thought to be restricted to inorganic semiconductors (Cornil *et al.*, 2001).

The molecule  of the title compound (Fig. 1) is centrosymmetric. An asymmetric unit comprises a half on the molecule. The inversion centre is located in the middle of C3—C3i bond. Conjugated molecular skeleton is nearly planar; mean deviation from the best least-square plane is 0.041 Å. The endocyclic bond angles on the long molecular axis are less than the normal 120° value (they vary from 116.42–117.52°) whereas that situated out of this long molecular axis are greater than 120° (in the range 120.73–122.43°). This result agrees with those obtained for polyphenyls (Robertson, 1961; Baudour, 1972; Domenicano *et al.*,  1975; Charbonneau & Delugeard, 1977). The two thiophene rings, phenyl rings and C≡C are coplanar. The crystal packing is dominated by van der Waals interactions.

### Experimental

The synthesis of 4,4'-bis-[2-(3,4-dibutyl-2-thienylethynyl)]biphenyl was performed as previously described (Liu *et al.*, 2005).

Yellow needles were grown from an ethanol/hexane solution by slow evaporation.

### Crystal data

**Table:**

C_40_H_46_S_2_	*V* = 849.7 (3) Å^3^
*M**_r_* = 590.89	*Z* = 1
Triclinic, *P*1	*F*_000_ = 318
*a* = 9.2040 (18) Å	*D*_x_ = 1.155 Mg m^−^^3^
*b* = 9.3640 (19) Å	Mo *K*α radiation λ = 0.71073 Å
*c* = 10.582 (2) Å	µ = 0.18 mm^−^^1^
α = 85.69 (3)º	*T* = 293 (2) K
β = 85.18 (3)º	Neddle, yellow
γ = 69.41 (3)º	0.62 × 0.40 × 0.07 mm

### Data collection

**Table:**

Rigaku R-AXIS RAPID IP diffractometer	3595 independent reflections
Radiation source: fine-focus sealed tube	2287 reflections with *I* > 2σ(*I*)
Monochromator: graphite	*R*_int_ = 0.0000
Detector resolution: 0.76 pixels mm^-1^	θ_max_ = 27.5º
*T* = 293(2) K	θ_min_ = 2.3º
Oscillation scans	*h* = 0→11
Absorption correction: empirical (using intensity measurements)(ABSCOR; Higashi, 1995)	*k* = −10→12
*T*_min_ = 0.805, *T*_max_ = 0.992	*l* = −13→13
3595 measured reflections	

### Refinement

**Table:**

Refinement on *F*^2^	Secondary atom site location: difference Fourier map
Least-squares matrix: full	Hydrogen site location: inferred from neighbouring sites
*R*[*F*^2^ > 2σ(*F*^2^)] = 0.067	H-atom parameters constrained
*wR*(*F*^2^) = 0.241	*w* = 1/[σ^2^(*F*_o_^2^) + (0.1672*P*)^2^ + 0.1626*P*] where *P* = (*F*_o_^2^ + 2*F*_c_^2^)/3
*S* = 1.07	(Δ/σ)_max_ < 0.001
3595 reflections	Δρ_max_ = 0.44 e Å^−^^3^
190 parameters	Δρ_min_ = −0.42 e Å^−^^3^
Primary atom site location: structure-invariant direct methods	Extinction correction: none

### Special details

**Table:**

Geometry. All e.s.d.'s (except the e.s.d. in the dihedral angle between two l.s. planes) are estimated using the full covariance matrix. The cell e.s.d.'s are taken into account individually in the estimation of e.s.d.'s in distances, angles and torsion angles; correlations between e.s.d.'s in cell parameters are only used when they are defined by crystal symmetry. An approximate (isotropic) treatment of cell e.s.d.'s is used for estimating e.s.d.'s involving l.s. planes.
Refinement. Refinement of *F*^2^ against ALL reflections. The weighted *R*-factor *wR* and goodness of fit *S* are based on *F*^2^, conventional *R*-factors *R* are based on *F*, with *F* set to zero for negative *F*^2^. The threshold expression of *F*^2^ > σ(*F*^2^) is used only for calculating *R*-factors(gt) *etc*. and is not relevant to the choice of reflections for refinement. *R*-factors based on *F*^2^ are statistically about twice as large as those based on *F*, and *R*- factors based on ALL data will be even larger.

### Fractional atomic coordinates and isotropic or equivalent isotropic displacement parameters (Å^2^)

**Table:**

	*x*	*y*	*z*	*U*_iso_*/*U*_eq_	
S1	0.34733 (8)	0.37884 (8)	0.02833 (7)	0.0512 (3)	
C1	−0.2000 (4)	0.5223 (4)	0.3588 (3)	0.0657 (9)	
H1A	−0.1334	0.5778	0.3558	0.079*	
C2	−0.3290 (4)	0.5588 (4)	0.4443 (3)	0.0615 (8)	
H2A	−0.3467	0.6388	0.4973	0.074*	
C3	−0.4314 (3)	0.4808 (3)	0.4534 (2)	0.0393 (5)	
C4	−0.4016 (4)	0.3655 (4)	0.3702 (4)	0.0750 (12)	
H4A	−0.4696	0.3116	0.3721	0.090*	
C5	−0.2733 (4)	0.3276 (5)	0.2840 (4)	0.0816 (13)	
H5A	−0.2573	0.2494	0.2295	0.098*	
C6	−0.1694 (3)	0.4041 (3)	0.2779 (2)	0.0439 (6)	
C7	−0.0335 (3)	0.3631 (3)	0.1922 (3)	0.0476 (6)	
C8	0.0798 (3)	0.3289 (3)	0.1211 (2)	0.0440 (6)	
C9	0.2138 (3)	0.2853 (3)	0.0369 (2)	0.0415 (6)	
C10	0.2565 (3)	0.1695 (3)	−0.0473 (2)	0.0387 (5)	
C11	0.3994 (3)	0.1568 (3)	−0.1180 (2)	0.0419 (6)	
C12	0.4587 (3)	0.2635 (3)	−0.0862 (3)	0.0497 (7)	
H12	0.5507	0.2720	−0.1235	0.060*	
C13	0.1620 (3)	0.0701 (3)	−0.0634 (3)	0.0463 (6)	
H13A	0.0948	0.0719	0.0126	0.056*	
H13B	0.2317	−0.0344	−0.0728	0.056*	
C14	0.4717 (3)	0.0371 (3)	−0.2152 (3)	0.0515 (7)	
H14A	0.4910	−0.0630	−0.1731	0.062*	
H14B	0.3969	0.0498	−0.2784	0.062*	
C15	0.0618 (3)	0.1215 (3)	−0.1791 (3)	0.0503 (6)	
H15A	0.1267	0.1352	−0.2527	0.060*	
H15B	0.0233	0.0412	−0.1960	0.060*	
C16	0.6214 (3)	0.0406 (3)	−0.2825 (3)	0.0498 (7)	
H16A	0.6965	0.0293	−0.2201	0.060*	
H16B	0.6025	0.1390	−0.3274	0.060*	
C17	−0.0748 (3)	0.2680 (4)	−0.1618 (3)	0.0575 (7)	
H17A	−0.0376	0.3445	−0.1336	0.069*	
H17B	−0.1465	0.2501	−0.0953	0.069*	
C18	0.6894 (3)	−0.0855 (4)	−0.3767 (3)	0.0604 (8)	
H18A	0.7095	−0.1838	−0.3314	0.073*	
H18B	0.6131	−0.0752	−0.4380	0.073*	
C19	−0.1626 (4)	0.3311 (4)	−0.2807 (3)	0.0680 (9)	
H19A	−0.2477	0.4233	−0.2623	0.102*	
H19B	−0.2019	0.2571	−0.3085	0.102*	
H19C	−0.0936	0.3526	−0.3464	0.102*	
C20	0.8373 (5)	−0.0823 (6)	−0.4465 (4)	0.0866 (13)	
H20A	0.8747	−0.1641	−0.5037	0.130*	
H20B	0.9140	−0.0944	−0.3866	0.130*	
H20C	0.8177	0.0135	−0.4937	0.130*	

### Atomic displacement parameters (Å^2^)

**Table:**

	*U*^11^	*U*^22^	*U*^33^	*U*^12^	*U*^13^	*U*^23^
S1	0.0432 (4)	0.0556 (4)	0.0568 (5)	−0.0203 (3)	0.0164 (3)	−0.0206 (3)
C1	0.0576 (17)	0.080 (2)	0.070 (2)	−0.0398 (16)	0.0352 (15)	−0.0323 (17)
C2	0.0596 (17)	0.0696 (18)	0.0622 (18)	−0.0334 (15)	0.0336 (14)	−0.0329 (15)
C3	0.0314 (11)	0.0457 (12)	0.0341 (11)	−0.0071 (9)	0.0110 (9)	−0.0058 (9)
C4	0.0615 (18)	0.088 (2)	0.090 (2)	−0.0447 (18)	0.0481 (18)	−0.055 (2)
C5	0.068 (2)	0.090 (2)	0.097 (3)	−0.0424 (19)	0.054 (2)	−0.062 (2)
C6	0.0326 (11)	0.0505 (14)	0.0415 (13)	−0.0084 (10)	0.0130 (10)	−0.0066 (10)
C7	0.0395 (13)	0.0532 (14)	0.0433 (14)	−0.0099 (11)	0.0122 (11)	−0.0087 (11)
C8	0.0372 (12)	0.0506 (14)	0.0393 (13)	−0.0113 (11)	0.0109 (10)	−0.0059 (10)
C9	0.0326 (11)	0.0477 (13)	0.0404 (12)	−0.0116 (10)	0.0126 (10)	−0.0065 (10)
C10	0.0321 (11)	0.0443 (12)	0.0366 (12)	−0.0116 (9)	0.0080 (9)	−0.0025 (9)
C11	0.0315 (11)	0.0488 (13)	0.0406 (13)	−0.0098 (10)	0.0118 (9)	−0.0082 (10)
C12	0.0360 (12)	0.0596 (15)	0.0526 (15)	−0.0182 (11)	0.0201 (11)	−0.0148 (12)
C13	0.0430 (13)	0.0452 (13)	0.0514 (14)	−0.0190 (11)	0.0110 (11)	−0.0048 (11)
C14	0.0385 (13)	0.0582 (16)	0.0552 (16)	−0.0142 (12)	0.0173 (11)	−0.0212 (13)
C15	0.0452 (13)	0.0580 (15)	0.0509 (15)	−0.0225 (12)	0.0100 (11)	−0.0140 (12)
C16	0.0374 (13)	0.0617 (16)	0.0442 (14)	−0.0113 (12)	0.0149 (11)	−0.0129 (12)
C17	0.0475 (15)	0.0641 (18)	0.0589 (17)	−0.0165 (13)	0.0028 (13)	−0.0116 (14)
C18	0.0478 (15)	0.077 (2)	0.0428 (15)	−0.0039 (14)	0.0070 (12)	−0.0187 (14)
C19	0.0546 (18)	0.080 (2)	0.068 (2)	−0.0214 (17)	−0.0071 (15)	−0.0038 (17)
C20	0.063 (2)	0.111 (3)	0.061 (2)	−0.003 (2)	0.0313 (17)	−0.018 (2)

### Geometric parameters (Å, °)

**Table:**

S1—C12	1.702 (3)	C13—H13A	0.9700
S1—C9	1.735 (3)	C13—H13B	0.9700
C1—C6	1.385 (4)	C14—C16	1.507 (3)
C1—C2	1.387 (4)	C14—H14A	0.9700
C1—H1A	0.9300	C14—H14B	0.9700
C2—C3	1.375 (4)	C15—C17	1.511 (4)
C2—H2A	0.9300	C15—H15A	0.9700
C3—C4	1.382 (4)	C15—H15B	0.9700
C3—C3^i^	1.490 (4)	C16—C18	1.527 (4)
C4—C5	1.387 (4)	C16—H16A	0.9700
C4—H4A	0.9300	C16—H16B	0.9700
C5—C6	1.377 (4)	C17—C19	1.519 (5)
C5—H5A	0.9300	C17—H17A	0.9700
C6—C7	1.435 (3)	C17—H17B	0.9700
C7—C8	1.193 (3)	C18—C20	1.502 (5)
C8—C9	1.414 (3)	C18—H18A	0.9700
C9—C10	1.382 (3)	C18—H18B	0.9700
C10—C11	1.428 (3)	C19—H19A	0.9600
C10—C13	1.507 (4)	C19—H19B	0.9600
C11—C12	1.369 (4)	C19—H19C	0.9600
C11—C14	1.514 (3)	C20—H20A	0.9600
C12—H12	0.9300	C20—H20B	0.9600
C13—C15	1.539 (4)	C20—H20C	0.9600
			
C12—S1—C9	91.29 (12)	C11—C14—H14A	108.5
C6—C1—C2	120.7 (3)	C16—C14—H14B	108.5
C6—C1—H1A	119.7	C11—C14—H14B	108.5
C2—C1—H1A	119.7	H14A—C14—H14B	107.5
C3—C2—C1	122.4 (3)	C17—C15—C13	113.7 (2)
C3—C2—H2A	118.8	C17—C15—H15A	108.8
C1—C2—H2A	118.8	C13—C15—H15A	108.8
C2—C3—C4	116.4 (2)	C17—C15—H15B	108.8
C2—C3—C3^i^	122.1 (3)	C13—C15—H15B	108.8
C4—C3—C3^i^	121.5 (3)	H15A—C15—H15B	107.7
C3—C4—C5	122.0 (3)	C14—C16—C18	112.4 (2)
C3—C4—H4A	119.0	C14—C16—H16A	109.1
C5—C4—H4A	119.0	C18—C16—H16A	109.1
C6—C5—C4	121.1 (3)	C14—C16—H16B	109.1
C6—C5—H5A	119.5	C18—C16—H16B	109.1
C4—C5—H5A	119.5	H16A—C16—H16B	107.9
C5—C6—C1	117.5 (2)	C15—C17—C19	114.3 (3)
C5—C6—C7	121.7 (2)	C15—C17—H17A	108.7
C1—C6—C7	120.8 (2)	C19—C17—H17A	108.7
C8—C7—C6	179.8 (4)	C15—C17—H17B	108.7
C7—C8—C9	178.8 (3)	C19—C17—H17B	108.7
C10—C9—C8	126.9 (2)	H17A—C17—H17B	107.6
C10—C9—S1	111.52 (17)	C20—C18—C16	113.2 (3)
C8—C9—S1	121.6 (2)	C20—C18—H18A	108.9
C9—C10—C11	112.0 (2)	C16—C18—H18A	108.9
C9—C10—C13	123.8 (2)	C20—C18—H18B	108.9
C11—C10—C13	124.2 (2)	C16—C18—H18B	108.9
C12—C11—C10	111.9 (2)	H18A—C18—H18B	107.7
C12—C11—C14	126.0 (2)	C17—C19—H19A	109.5
C10—C11—C14	122.0 (2)	C17—C19—H19B	109.5
C11—C12—S1	113.24 (18)	H19A—C19—H19B	109.5
C11—C12—H12	123.4	C17—C19—H19C	109.5
S1—C12—H12	123.4	H19A—C19—H19C	109.5
C10—C13—C15	112.8 (2)	H19B—C19—H19C	109.5
C10—C13—H13A	109.0	C18—C20—H20A	109.5
C15—C13—H13A	109.0	C18—C20—H20B	109.5
C10—C13—H13B	109.0	H20A—C20—H20B	109.5
C15—C13—H13B	109.0	C18—C20—H20C	109.5
H13A—C13—H13B	107.8	H20A—C20—H20C	109.5
C16—C14—C11	115.3 (2)	H20B—C20—H20C	109.5
C16—C14—H14A	108.5		
			
C12—C11—C10—C9	−0.6 (4)	C4—C5—C6—C1	−1.5 (1)
C12—S1—C9—C10	0.1 (8)	C1—C2—C3—C4	−1.6 (0)
C10—C11—C12—S1	0.5 (1)	C5—C4—C3—C2	1.5 (0)
C11—C12—S1—C9	−0.2 (0)	C5—C6—C1—C2	1.4 (2)
C11—C10—C9—S1	0.4 (9)	C9—C8—C7—C6	93.5 (2)
C11—C10—C9—C8	−179.9 (8)	C10—C9—C8—C7	24.6 (9)
C12—S1—C9—C8	−179.7 (4)	S1—C9—C8—C7	−155.8 (2)
C7—C6—C5—C4	177.9 (5)	C5—C6—C7—C8	−117.5 (4)
C7—C6—C1—C2	−178.0 (5)	C1—C6—C7—C8	61.9 (0)
C6—C5—C4—C3	0.0 (4)	C4—C3—C3—C4	90.0 (0)
C6—C1—C2—C3	0.1 (5)		

Symmetry codes: (i) −*x*−1, −*y*+1, −*z*+1.
